# The Most Recent Oncologic Emergency: What Emergency Physicians Need to Know About the Potential Complications of Immune Checkpoint Inhibitors

**DOI:** 10.7759/cureus.1774

**Published:** 2017-10-13

**Authors:** Duncan Simmons, Eddy Lang

**Affiliations:** 1 Faculty of Medicine and Dentistry, University of Alberta; 2 Emergency Medicine, University of Calgary

**Keywords:** immune checkpoint inhibitor, ctla-4, pd-1, pd-l1, immune-related adverse event, oncology, immunotherapy, emergency medicine, emergency department, review

## Abstract

Immune checkpoint inhibitors targeting cytotoxic T-lymphocyte associated protein 4 (CTLA-4) and programmable cell death protein 1 (PD-1)/PD-L1 have shown antitumor activity in cancers such as melanoma, non-small cell lung cancer, renal cell carcinoma, and urothelial cancer. Certain checkpoint inhibitors have been approved for use in Canada, and are becoming a mainstay in the treatment of melanoma and other malignancies. These drugs have a unique side effect profile and are known to cause immune-related adverse events (irAEs). These adverse events often appear to originate from an infectious etiology, when in fact they result from the enhanced immune response caused by immune checkpoint therapy. IrAEs are primarily treated with corticosteroids, which suppress the overactive immune response that is secondary to the treatment. IrAEs can occur in any organ system, but adverse events in the skin, gastrointestinal, endocrine, and pulmonary systems are among the most common. As an emergency physician, one must be familiar with these drugs and their adverse events in order to identify patients presenting with irAE and treat them accordingly. This paper provides a brief introduction to immune checkpoint inhibitors, discusses the most common irAEs relevant to emergency physicians, and gives suggestions on how to manage patients presenting to the emergency department (ED) suffering from irAEs.

## Introduction and background

Immune checkpoint inhibitors are novel cancer therapeutics that enhance the anti-tumor immune response to various malignancies. These drugs work by blocking inhibitory immune checkpoints. Immune checkpoints are pathways that serve to either up-regulate or down-regulate the immune response. In a healthy individual, inhibitory immune checkpoint molecules promote self-tolerance, reducing autoimmunity. However, in an individual with cancer, they impair the immune-mediated clearance of tumor cells. Tumor cells are able to use inhibitory checkpoint molecules as a means to evade immune clearance, by upregulating their expression on the tumor cell surface [1].

There are many different immune checkpoint pathways being investigated as therapeutic targets. Currently, successful therapies have been developed that inhibit the cytotoxic T-lymphocyte associated protein 4 (CTLA-4) pathway and the programmable cell death protein 1 (PD-1)/PD-L1 pathway. CTLA-4 is an immune checkpoint molecule expressed on T cells that, when bound to its ligands B7-1 or B7-2, impairs the activation of T cells [1]. PD-1 is an immune checkpoint molecule expressed on activated T cells, B cells, and NK cells. When PD-1 interacts with its ligands, PD-L1 or PD-L2, it down-regulates the effector response of these cells in peripheral tissues. The predominant effect of PD-1 inhibition is due to enhanced T cell effector function, however, enhanced antibody production and NK cell activity also likely play a role [1]. Inhibiting either the CTLA-4 or PD-1 checkpoint pathway results in an enhanced anti-tumor immune response. Monoclonal antibodies (mAbs) to these molecules have been developed. Currently, in Canada, four drugs have been approved for use in treating multiple cancers, namely ipilimumab, nivolumab, pembrolizumab, and atezolizumab. Ipilimumab, the first immune checkpoint inhibitor approved for use, is a mAb that targets and inhibits CTLA-4. Nivolumab and pembrolizumab are mAbs that target and inhibit PD-1, and atezolizumab targets and inhibits PD-L1. Table 1 provides a list of these agents, as well as other notable immune checkpoint inhibitors that are not yet approved in Canada. These drugs are typically administered in a regimen consisting of multiple doses over the span of months. Ipilimumab is given every three weeks for a total of four doses. Nivolumab is given every two weeks until the disease progresses or the patient can no longer tolerate the drug [2]. Pembrolizumab and atezolizumab are given every three weeks until the disease progresses or the patient can no longer tolerate the drug [3-4].

**Table 1:** Notable immune checkpoint inhibitors ^1^Health Canada-approved immune checkpoint inhibitors

Checkpoint molecule targeted	Generic name	Trade name
CTLA-4	Ipilimumab^1^	Yervoy
	Tremelimumab	-
PD-1	Nivolumab^1^	Opdivo
	Pembrolizumab^1^	Keytruda
PD-L1	Atezolizumab^1^	Tecentriq
	Avelumab	Bavencio
	Durvalumab	Imfinzi

Ipilimumab was the first drug to demonstrate improved overall survival in patients with metastatic melanoma. In a Phase 3 randomized controlled trial comparing ipilimumab to control treatment in patients with metastatic melanoma, control treatment resulted in a median overall survival, and two-year survival of 6.4 months and 13.7% respectively. Treatment with ipilimumab improved median overall survival, and two-year survival to 10.1 months and 23.5% respectively [5]. Subsequent randomized controlled trials involving ipilimumab and the other immune checkpoint inhibitors demonstrated substantial antitumor activity, leading to their approval in Canada for the treatment of metastatic melanoma (ipilimumab, nivolumab, pembrolizumab) [3, 5-6], non-small cell lung cancer (nivolumab, pembrolizumab) [7-9], renal cell carcinoma (nivolumab) [10], squamous cell cancer of the head and neck (nivolumab) [11], and urothelial carcinoma (atezolizumab) [4]. The combination of ipilimumab and nivolumab is also approved for use in patients with metastatic melanoma [2, 12], and treatment trends are moving towards the increased use of combination therapy. A very recent study of patients with advanced melanoma showed that treatment with ipilimumab and nivolumab combination therapy or nivolumab monotherapy resulted in longer progression-free survival than ipilimumab monotherapy. Although the study was not powered to compare the two nivolumab-containing groups, overall survival was numerically higher at three years in the combination therapy group than in the group treated with nivolumab monotherapy [13]. Novel immune checkpoint inhibitors targeting other immune checkpoint molecules including LAG-3, TIM-3, GITR, and OX40 are currently being investigated in clinical trials, and it is likely that there will soon be more agents available for use in a wider variety of malignancies, with a greater number of combination therapies available.

## Review

Immune checkpoint inhibitors have an adverse event profile distinct from that of conventional chemotherapy. These drugs result in toxicities known as immune-related adverse events (irAEs), which occur secondary to the enhanced immune-response that the drugs elicit, and can occur in any organ system. However, they most commonly affect the skin, gastrointestinal, endocrine, and pulmonary systems. Notable examples of irAEs in these organ systems are summarized in Table 2. Toxicities to other systems that are less likely to result in patients presenting to the emergency department (ED), or are uncommon, can include hepatic, renal, neurologic, hematologic, ocular, cardiovascular, and rheumatic irAEs, among others [14-16]. The irAE profile is similar between the different checkpoint inhibitors. However, CTLA-4 inhibitors have shown to cause irAEs more frequently than PD-1 inhibitors, and combination therapy results in a greater severity and frequency of irAEs than either monotherapy [17]. It is important for physicians to be aware that irAEs can occur at any time–from the outset of treatment, during treatment, or after treatment has been discontinued [18]. A pooled study of nivolumab monotherapy found that, although 85% of irAEs began within the first 16 weeks of therapy, they can occur more than a year after initiating treatment [19]. This stands in contrast to chemotherapy, where the timing of adverse events secondary to treatment is more predictable–for example, neutropenic related events, which are known to occur most often during the first cycle of chemotherapy [20].

**Table 2:** Notable immune-related adverse events by system

Organ system	Immune-related adverse event
Skin	Pruritus, maculopapular rash, papulopustular rash, Sweet’s syndrome, vitiligo, Stevens-Johnson syndrome, toxic epidermal necrolysis
Gastrointestinal	Diarrhea, colitis
Endocrine	Hypophysitis, hypothyroidism, Grave’s disease, thyroid storm, insulin-dependent diabetes, adrenal insufficiency
Pulmonary	Pneumonitis

**Figure 1:**
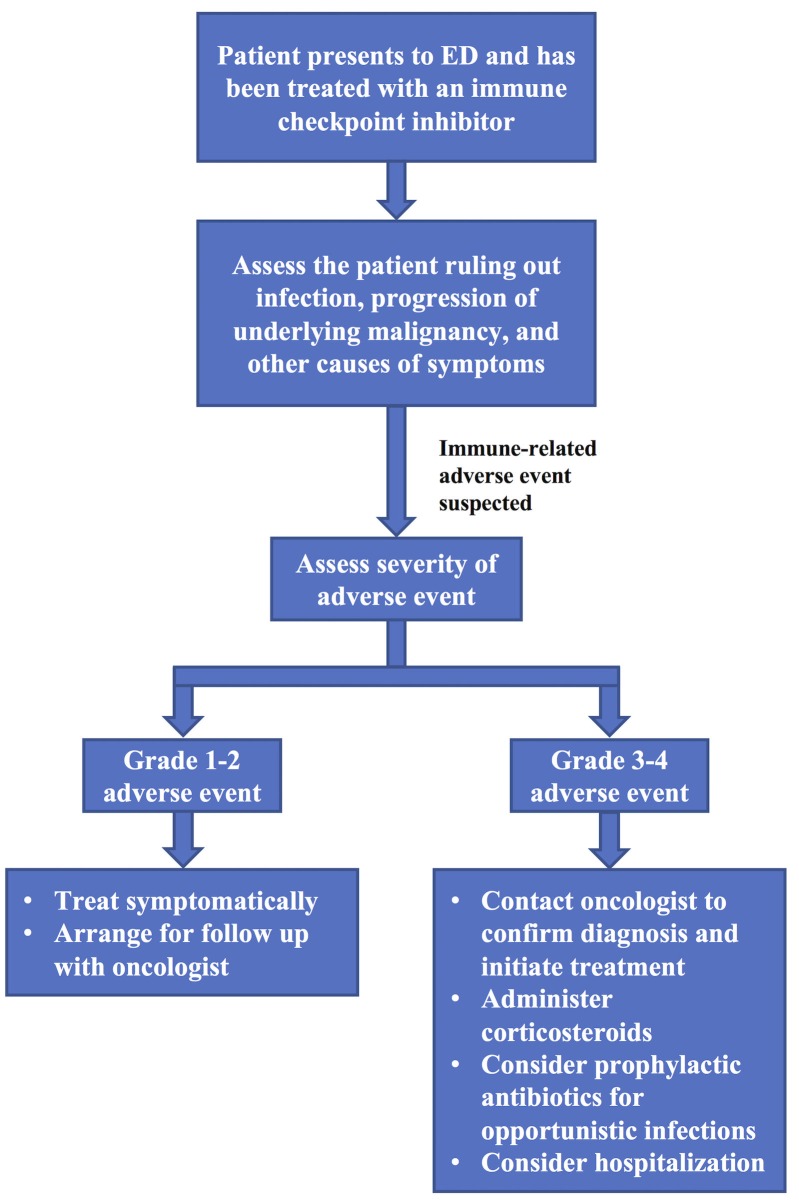
Emergency department management of a patient on an immune checkpoint inhibitor ED = emergency department

Figure 1 provides a basic overview of how to manage a patient on an immune checkpoint inhibitor presenting to the ED. Guidelines have been published outlining the management of patients suffering from irAEs [21-22], however, they are oriented towards oncologists and may not be devised in a way that is optimized for the emergency medicine context. Systemic corticosteroids are the primary therapy indicated for the treatment of irAE, though in less severe cases symptomatic treatment can be used; and in more severe cases refractory to corticosteroids, other immunomodulators, such as infliximab or mycophenolate mofetil might be considered [16, 22]. Although, in theory, there is a concern that the use of corticosteroids would reduce the efficacy of immune checkpoint therapy by reducing the immune response to tumor cells, to date studies have suggested that corticosteroid use does not affect the antitumor response [19, 23-25]. Before diagnosing and treating a patient with an irAE, it is important to rule out infection and progression of the underlying malignancy as the cause of a patients’ symptoms. Due to the effectiveness of these drugs, they are being prescribed more commonly, and ED presentation of adverse events will increase in frequency. Because of this, it is important that emergency physicians familiarize themselves with immune checkpoint inhibitors and the irAEs that can result from their use, and understand how to treat these irAEs. This will help to ensure that irAEs are identified and treated early, as early treatment is crucial to limiting their severity and duration [18-19]. Treatment is quite effective as 85-100% of grade 3 to grade 4 irAEs resolve with proper treatment. The exception to this is endocrine irAEs, which generally require long-term hormone replacement therapy [15, 22].

Site-specific immune-related adverse events

Skin

Skin toxicities are the most common, and often earliest irAEs experienced by those on immune checkpoint therapy [14, 17]. Toxicity is more common in combination therapy than monotherapy–skin irAE of any grade have been observed in 62% of patients on nivolumab plus ipilimumab combination therapy, compared with 56% of patients on ipilimumab monotherapy and 46% of patients on nivolumab monotherapy [13]. Skin irAEs can present as pruritus, maculopapular rash, Sweet’s syndrome, and vitiligo [17, 26]. More severe and potentially fatal skin toxicities can also occur, as instances of Stevens-Johnson syndrome and toxic epidermal necrolysis have been described–if either of these complications occurs, immune checkpoint therapy should likely be discontinued permanently [17]. It is important to rule out non-inflammatory causes of these skin conditions, or progression of the patient’s underlying malignancy before diagnosing and treating them as an irAE.

Most instances of skin irAE will be less-severe grade 1-2 rashes (Table 3), and these patients will likely not present to the ED. If they do, they can continue their immune checkpoint therapy, and be treated symptomatically with topical corticosteroids (e.g. betamethasone 0.1%) and oral antihistamines as needed. It is necessary to ensure that the patient is aware that, if their symptoms worsen or persist for greater than one to two weeks after treatment, they should follow up with their oncologist, as this may necessitate the use of oral or intravenous corticosteroids [21, 27]. Approximately 6% of patients on combination therapy, 3% of patients on ipilimumab monotherapy, and 2% of patients on nivolumab monotherapy will experience grade 3-4 skin irAE [13]. With grade 3-4 rashes, immune checkpoint therapy should be delayed, 1-2 mg/kg/day IV methylprednisolone (or oral equivalent) should be administered, a dermatology consult should be scheduled, and the patient may need to be admitted to the hospital depending on the severity of the irAE. Follow up with the patient’s oncologist should be scheduled to taper the corticosteroids and decide whether or not treatment should be resumed [21].

**Table 3:** Grading of common immune-related adverse events As per the National Cancer Institute’s Common Terminology Criteria for Adverse Events (CTCAE), version 4 [28]

Adverse event	Grade 1	Grade 2	Grade 3	Grade 4
Rash	Covering <10% body surface area with or without associated symptoms	Covering 10-30% body surface area with or without associated symptoms	Covering >30% body surface area with or without associated symptoms	–
Diarrhea	<4 stools/day above baseline; mild increase in ostomy output	4-6 stools/day above baseline; moderate increase in ostomy output	>7 stools/day above baseline; hospitalization indicated; incontinence; severe increase in ostomy output	Life-threatening consequences; urgent intervention indicated
Colitis	Asymptomatic; intervention not indicated	Abdominal pain; mucus or blood in stool	Severe abdominal pain; changes in bowel habits; medical intervention indicated; peritoneal signs	Life-threatening consequences; urgent intervention indicated
Pneumonitis	Asymptomatic; intervention not indicated	Symptomatic; medical intervention indicated	Severe symptoms; oxygen indicated	Life-threatening respiratory compromise; urgent intervention indicated

Gastrointestinal

The notable gastrointestinal irAEs resulting from immune checkpoint therapy are diarrhea and colitis, both of which are observed more often with combination therapy, and least often with PD-1 monotherapy. One study found that diarrhea of any grade occurs in approximately 45% of patients on combination therapy, 34% of patients on ipilimumab monotherapy, and 21% of patients on nivolumab monotherapy, with grade 3-4 diarrhea occurring in approximately 9% of patients on combination therapy, 6% of patients on ipilimumab monotherapy, and 3% of patients on nivolumab monotherapy. In the same study, colitis of any grade was found to occur in approximately 13% of patients on combination therapy, 11% of patients on ipilimumab monotherapy, and 2% of patients on nivolumab monotherapy, with grade 3-4 colitis occurring in 8% of patients on combination therapy, 8% of patients on ipilimumab monotherapy, and 1% of patients on nivolumab monotherapy [13]. A rare but emergent gastrointestinal irAE is bowel perforation secondary to colitis–this is observed in ≤1% of patients, but it is potentially life-threatening [22, 27, 29]. It is important to still consider other causes for gastrointestinal symptoms in patients on immune checkpoint inhibitors, such as infection or progression of the patient’s underlying malignancy. However, if these causes are ruled out, symptoms should be managed as irAEs.

Grade 1 diarrhea should be managed symptomatically with rehydration and antimotility agents. Grade 2 diarrhea or colitis can also be managed symptomatically, but immune checkpoint treatment should be delayed until the symptoms improve to grade 1. Patients should be advised to contact their oncologist if grade 2 symptoms persist for 5-7 days after symptomatic treatment, as they may benefit from corticosteroids. If the patient is suffering from grade 3-4 diarrhea or colitis immune checkpoint therapy should be discontinued, and 1-2 mg/kg/day IV methylprednisolone (or equivalent) should be given. One should consider also administering antibiotics for the prophylactic treatment of opportunistic infections that might arise while on corticosteroids. Depending on the severity of their symptoms, and concern of bowel perforation, these patients may need to receive a colonoscopy and/or be admitted to the hospital. Patients should follow up with their oncologist to determine when to taper their corticosteroids and to consider the addition of infliximab to the treatment regimen if their symptoms are refractory to corticosteroid therapy [21-22, 30-31].

Endocrine

Endocrine irAEs are likely the most difficult to diagnose, therefore it is important to keep these complications in mind when a patient on an immune checkpoint inhibitor presents to the ED with non-specific symptoms. Common symptoms associated with endocrine irAE include fatigue, headache, and nausea–symptoms which are associated with and might be attributed to a patient’s underlying malignancy [15, 17, 32]. Hypophysitis, hypothyroidism, and hyperthyroidism are the irAE most commonly experienced by patients on checkpoint therapy. In one study hypothyroidism, hyperthyroidism, and hypophysitis of any grade was observed in 17%, 11%, and 7% of patients on combination therapy, 11%, 4%, and 1% of patients on nivolumab monotherapy, and 5%, 1%, and 4% of patients on ipilimumab monotherapy, respectively [13]. Hypophysitis is a rare diagnosis, but due to the increasing use of immune checkpoint inhibitors, it will likely become increasingly common. Hypophysitis can result in low levels of some or all of the hormones produced by the pituitary. The most common symptoms associated with hypophysitis are headache, fatigue, and arthralgia. Hypophysitis can be diagnosed by the presence of these symptoms; magnetic resonance imaging showing enlargement and enhancement of the pituitary, and biochemical testing demonstrating pituitary dysfunction (low thyrotropin-releasing hormone and/or adrenocorticotrophic hormone) [17, 33]. Other endocrinopathies associated with immune checkpoint inhibitors include thyroiditis, Grave’s disease, thyroid storm, insulin-dependent diabetes mellitus, and adrenal insufficiency [15, 34-35]. Adrenal crisis is the most serious and life-threatening endocrine irAE, and requires immediate management with corticosteroids [14]. Adrenal crisis can occur secondary to hypophysitis, or due to primary adrenal insufficiency [33, 36].

Unlike irAEs in other organ systems which generally resolve completely after appropriate therapy is administered, endocrine irAEs most often require permanent hormone replacement therapy [15]. If an endocrine irAE is suspected, and other potential causes have been ruled out, consider conducting visual field testing, imaging the pituitary, and consulting endocrinology. In symptomatic patients suffering from endocrine irAEs in which an abnormal lab value and/or pituitary scan has been identified, immune checkpoint therapy should be delayed and 1-2 mg/kg/day methylprednisolone IV (or oral equivalent), as well as any additional appropriate hormone therapy, should be administered. The patient may need to be admitted to the hospital depending on the severity of their symptoms. If the patient is hypotensive, severely dehydrated, or in shock, and an adrenal crisis is suspected, delay checkpoint therapy, IV fluids and a stress dose of IV corticosteroids (with mineralocorticoid activity) should be administered, and endocrinology should be consulted. Before treating as an adrenal crisis, it is necessary to ensure that sepsis is ruled out as a possible cause of these symptoms [21-22].

Pulmonary

The main pulmonary irAE of interest is pneumonitis. Pneumonitis is potentially fatal and was the cause of three deaths in early trials of PD-1 inhibitors [37]. Pneumonitis occurs in <10% of patients on checkpoint therapy, but it is more commonly observed with PD-1 inhibitor therapy than CTLA-4 inhibitor therapy and is more common in combination therapy than either monotherapy [3]. In a pooled analysis investigating the irAEs of PD-1 inhibitors, pneumonitis was the most common irAEs leading to discontinuation of therapy [26]. Pneumonitis can occur at any time; however, it most often occurs later than irAEs in other organ systems and often can occur months after treatment is initiated [38]. Patients on immune checkpoint therapy presenting with new pulmonary symptoms including symptoms similar to an upper respiratory infection, cough, hypoxia, or shortness of breath should be imaged with a chest computerized tomography scan to evaluate for pneumonitis [14]. Rarely, sarcoidosis has also been observed in patients on immune checkpoint therapy; however, it is assessed and treated similarly to pneumonitis [15].

For individuals with grade 2 pneumonitis, immune checkpoint therapy should be delayed, 1 mg/kg/day IV methylprednisolone (or oral equivalent) should be administered, and pulmonology and/or infectious disease should be consulted. Depending on the severity of the patient’s symptoms, hospitalization should be considered and bronchoscopy or lung biopsy may need to be considered to evaluate for infectious etiology. These patients should be informed to follow up with their oncologist in order to determine when to taper their corticosteroids and to return to the ED if their symptoms worsen or do not improve after two weeks. Patients presenting with grade 3-4 pneumonitis should be hospitalized, have their immune checkpoint therapy discontinued, and receive 2-4 mg/kg/day IV methylprednisolone (or IV equivalent). These patients may also require prophylactic antibiotics, bronchoscopy, and/or lung biopsy [21-22].

## Conclusions

Immune checkpoint inhibitors have shown promise in the treatment of multiple malignancies. Because of this, these drugs are becoming more prevalent, and their increased use will result in more patients presenting to the ED suffering from irAE. Emergency physicians must be aware of the existence of these drugs, their adverse event profiles, and know how to manage these adverse events in the ED. Although the most common and serious irAE affect the skin, gastrointestinal, endocrine, and pulmonary systems, irAE from immune checkpoint inhibitors can manifest in any organ system. When a patient presents to the emergency department and an immune checkpoint inhibitor is on their medication list, it is important that ED physicians maintain a high index of suspicion for irAEs.
